# CAF-1 Is Essential for Heterochromatin Organization in Pluripotent Embryonic Cells

**DOI:** 10.1371/journal.pgen.0020181

**Published:** 2006-11-03

**Authors:** Martin Houlard, Soizik Berlivet, Aline V Probst, Jean-Pierre Quivy, Patrick Héry, Geneviève Almouzni, Matthieu Gérard

**Affiliations:** 1 Epigenetic Regulation and Cancer Group, Service de Biologie Moléculaire Systémique, Département de Biologie Joliot-Curie, Direction des Sciences du Vivant, Commissariat à l'Energie Atomique, CEA Saclay, Gif-sur-Yvette, France; 2 Section de Recherche, Institut Curie, UMR218 du Centre National de la Recherche Scientifique, Paris, France; The Babraham Institute, United Kingdom

## Abstract

During mammalian development, chromatin dynamics and epigenetic marking are important for genome reprogramming. Recent data suggest an important role for the chromatin assembly machinery in this process. To analyze the role of chromatin assembly factor 1 (CAF-1) during pre-implantation development, we generated a mouse line carrying a targeted mutation in the gene encoding its large subunit, p150CAF-1. Loss of p150CAF-1 in homozygous mutants leads to developmental arrest at the 16-cell stage. Absence of p150CAF-1 in these embryos results in severe alterations in the nuclear organization of constitutive heterochromatin. We provide evidence that in wild-type embryos, heterochromatin domains are extensively reorganized between the two-cell and blastocyst stages. In p150CAF-1 mutant 16-cell stage embryos, the altered organization of heterochromatin displays similarities to the structure of heterochromatin in two- to four-cell stage wild-type embryos, suggesting that CAF-1 is required for the maturation of heterochromatin during preimplantation development. In embryonic stem cells, depletion of p150CAF-1 using RNA interference results in the mislocalization, loss of clustering, and decondensation of pericentric heterochromatin domains. Furthermore, loss of CAF-1 in these cells results in the alteration of epigenetic histone methylation marks at the level of pericentric heterochromatin. These alterations of heterochromatin are not found in p150CAF-1-depleted mouse embryonic fibroblasts, which are cells that are already lineage committed, suggesting that CAF-1 is specifically required for heterochromatin organization in pluripotent embryonic cells. Our findings underline the role of the chromatin assembly machinery in controlling the spatial organization and epigenetic marking of the genome in early embryos and embryonic stem cells.

## Synopsis

Chromatin is the support of our genetic information. It is composed of numerous repeated units called nucleosomes, in which DNA wraps around a core of histone proteins. Modifications in the composition and biochemical properties of nucleosomes play major roles in the regulation of genome function. Such modifications are termed “epigenetic” when they are inherited across cell divisions and confer new information to chromatin, in addition to the genetic information provided by DNA. It is usually believed that during genome replication, the basic chromatin assembly machinery builds up “naïve” nucleosomes, and, in a subsequent step, nucleosomes are selectively modified by a series of enzymes to acquire epigenetic information. Here, the authors studied the role of a basic chromatin assembly factor (CAF-1) in mouse embryonic stem cells and early embryos. Surprisingly, they show that CAF-1 confers epigenetic information to specific genomic regions. In addition, this study revealed that CAF-1 is required for the proper spatial organization of chromosomes in the nucleus. This new knowledge may contribute to better understanding the role of chromatin in the maintenance of embryonic stem cell identity and plasticity.

## Introduction

During mouse pre-implantation development, the genome undergoes a series of major epigenetic changes required for embryonic gene expression, the maintenance of totipotency, and the first differentiation events [1,2]. While many studies have established the importance of DNA methylation in epigenetic reprogramming, recent data point to a crucial role of chromatin in this process [3–5]. Yet we know very little about the role of histone modifying enzymes, chromatin remodeling factors, and histone chaperones during pre-implantation development, or in stem cells derived from early embryos [6]. A subset of identified histone chaperones and chromatin remodeling complexes can collaborate to promote nucleosome assembly in vitro and are therefore in a strategic position to control chromatin assembly and maturation during development [7,8]. Among histone chaperones, chromatin assembly factor 1 (CAF-1) is a three-subunit (p150, p60, and p48) complex which promotes histone H3 and H4 deposition onto newly synthesized DNA during replication or DNA repair [9,10]. Specifically, CAF-1 deposits the histone H3 variant H3.1 into chromatin, in a pathway coupled to DNA synthesis, whereas a second histone chaperone, HIRA, is involved in the deposition of the H3.3 variant in a DNA synthesis independent pathway [11,12]. In addition to this specific chromatin assembly activity, CAF-1 interacts with several proteins present in heterochromatin, including heterochromatin protein 1 (HP1) and MBD1, a methyl-CpG binding domain protein that recruits histone deacetylase and repressive histone methyltransferase activities [13–15]. Furthermore, p150CAF-1 is required to ensure a replication-specific pool of HP1 molecules at replication sites in pericentric heterochromatin during mid-late S phase [16]. Taken together, these data suggest roles for CAF-1 in the formation of heterochromatin and in the heritability of epigenetic traits. While the function of this evolutionary conserved histone chaperone has been studied extensively biochemically, we still lack information concerning its importance during early development in mammals and in pluripotent cells such as embryonic stem (ES) cells. Here, we have analyzed the importance of CAF-1 during early mouse development by genetic ablation and in ES cells by depletion using RNA interference (RNAi). We show that CAF-1 is essential for viability in early mouse embryos and ES cells. We provide evidence that CAF-1 is required for the spatial organization and epigenetic marking of heterochromatin domains in pluripotent embryonic cells.

## Results

**Figure 1:**
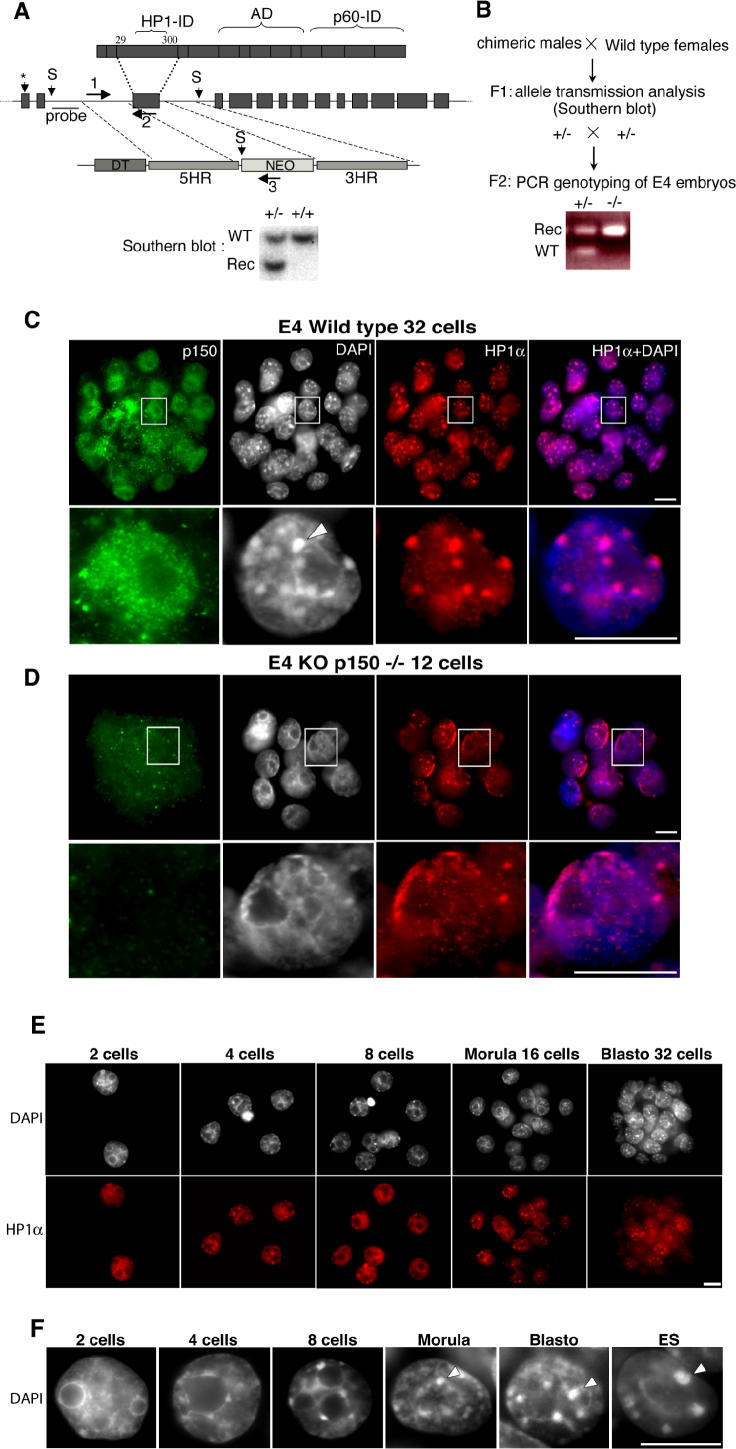
Loss of p150CAF-1 Function Leads to Early Developmental Arrest and Alteration of Heterochromatin Organization (A) Generation of *Chaf1a* ^+/*−*^ mice by homologous recombination in ES cells. (Top) Scheme of the p150CAF1 protein indicating the interacting domains (ID) for HP1 and p60CAF-1, and the acidic domain (AD). (Middle) Structure of the murine *Chaf1a* gene. Blocks and lines represent exons and introns, respectively. A star indicates the translation initiation site. Below is shown the *Chaf1a* targeting vector, which includes genomic DNA homology regions (5 HR and 3 HR), the diphtheria toxin (DT), and neomycin selection genes. Recombined (Rec) ES cells and mice were identified by Southern blot using *Sca*I (S) and the indicated probe. (B) Breeding strategy used for the production of E4 *Chaf1a* ^−/*−*^ embryos. Genotyping was performed by PCR using the three oligonucleotide primers indicated in (A) (1, 2, and 3). An example of the result of a genotyping experiment is shown. (C) Immunodetection of p150CAF-1 (green) and HP1α (red) in E4 embryos derived from *Chaf1a* ^+/*−*^ intercrosses. Because *Chaf1a* ^+/+^ and *Chaf1a* ^+/*−*^ embryos both stain positively for the presence of p150CAF-1, they are both designated as wild-type. p150CAF-1 expression, which indicates ongoing S phase, can be detected in most cells within the wild-type blastocyst (upper panel). The lower panel shows a 12-cell embryo labeling negatively for p150CAF-1. Only nonspecific background labeling can be observed. In each panel, the right-hand image shows the merge between the HP1α fluorescence and DAPI-stained DNA in blue. Pink color indicates the association of HP1 with DAPI-dense material. The bottom of each panel shows the magnification of a nucleus selected from the above embryo (white square). The arrowhead indicates the typical heterochromatin foci revealed by DAPI and HP1α staining in wild-type embryos. These foci are not visible in *Chaf1a* ^−/−^ embryos. DAPI and HP1α staining are diffuse within the nucleus of p150CAF-1-depleted embryos, with enrichment at the nuclear periphery, revealing abnormal heterochromatin organization. Scale bars represent 10 μm. (E) Wild-type embryos isolated at embryonic day 2 (E2, two cells), E2.5 (four cells), E3 (eight cells), E3.5 (16 cells), and E4 (32 cells) are shown. Heterochromatin was monitored by DAPI staining (upper panel) and HP1 immunolabeling (in red, lower panel). (F) Magnification of a nucleus (DAPI-stained) representative of each stage. The right-hand panel shows the nucleus of an ES cell. Scale bar represents 10 μm.

We used gene targeting in ES cells to delete exon 3 in the *Chaf1a* gene, which encodes p150CAF-1 (Figure 1A). *Chaf1a* ^+/*−*^ mice were born at a Mendelian frequency, were of normal size and weight, displayed no obvious abnormalities, and were fertile. Crossing heterozygous mice failed to generate viable newborn *Chaf1a* ^−/*−*^ mice. Furthermore, no homozygous *Chaf1a* ^−/*−*^ embryos were detected at any of the post-implantation stages. However, we could detect *Chaf1a* ^−/*−*^ embryos at embryonic day 4 (E4) using a PCR strategy (Figure 1B). They represent only 10% of E4 embryos obtained from *Chaf1a* ^+/*−*^ mice intercrosses, suggesting that more than half of the homozygous mutant embryos had degenerated before this stage. Moreover, mutant embryos contained only eight to 16 cells at the E4 stage, instead of 32 cells observed for wild-type or heterozygous blastocysts (Figure 1C and 1D). Further analysis by light microscopy using immunofluorescence (IF) allowed us to compare wild-type and p150CAF-1-depleted embryos. A loss of p150CAF-1 staining could not be detected before the eight-cell stage (unpublished data) suggesting that maternally contributed protein is present in the embryo up to the four-cell stage. Thus, depletion of p150CAF-1 protein from this stage allows a maximum of two additional cell divisions before developmental arrest.

Strikingly, we found that the nuclear organization of heterochromatin appeared abnormal in the nuclei of *Chaf1a* ^−/*−*^ embryos. Pericentric heterochromatin, the major component of constitutive heterochromatin, can easily be visualized in interphase nuclei by the fluorochrome DAPI and by immunostaining with HP1α [16,17–19]. In wild-type blastocysts and in mouse somatic cells, pericentric heterochromatin domains cluster together and form higher-order chromatin structures called chromocenters [20], which are visualized as DAPI-dense foci (Figure 1C). These structures were not detected in *Chaf1a* ^−/*−*^ embryos (Figure 1D). Since chromatin architecture and higher-order structures are important for genome function [21], we decided to characterize this phenotype in more detail. For this purpose, we examined the status of heterochromatin organization between the two-cell and blastocyst stages. In two-cell stage wild-type embryos, DAPI staining is diffuse and fibrillar, with regions of higher density around the nucleolar precursor bodies (Figure 1E). Heterochromatin domains are progressively assembled into DAPI-dense foci between the four-cell and 32-cell blastocyst stages (Figure 1F). Localization of HP1α confirmed that the structures visualized by DAPI staining correspond to constitutive heterochromatin (Figure 1E). Thus, the nuclear organization of heterochromatin is dramatically modified during pre-implantation development, between the two-cell and blastocyst stages. In *Chaf1a* ^−/*−*^ E4 embryos, which are arrested between the eight- and 16-cell stage, DAPI-dense foci were barely detectable (Figure 1D). Instead, DAPI staining was diffuse within the nucleus, with regions of higher density around the nucleoli and at the periphery of the nuclei (Figure 1D). Localization of HP1α showed a diffuse pattern similar to the DAPI, with some enrichment at the nuclear periphery, and around the nucleoli (Figure 1D). This abnormal organization of heterochromatin in *Chaf1a* ^−/*−*^ embryos is reminiscent of the heterochromatin organization found in two- to four-cell stage wild-type embryos (compare Figure 1D and 1F). These data evidence a key role for p150CAF-1 during pre-implantation development, and reveal that this protein is required for the proper 3-D organization of heterochromatin within embryonic cell nuclei.

**Figure 2:**
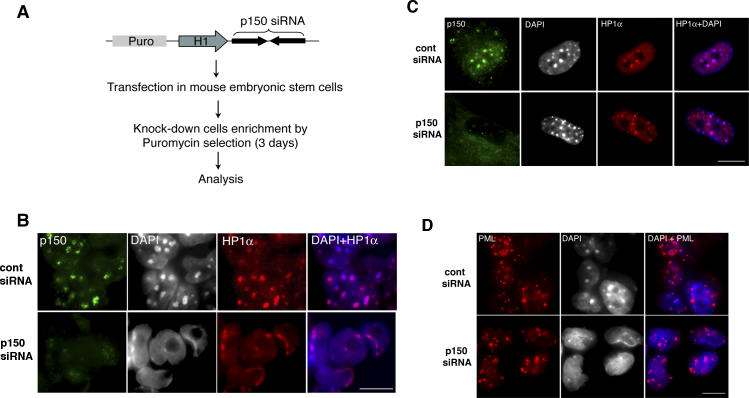
Depletion of p150CAF-1 in ES Cells Results in a Severe Alteration of Heterochromatin Organization (A) Strategy used to deplete p150CAF-1 by RNAi in ES cells. The siRNA expression vector includes a puromycin selection cassette (Puro), the mouse H1 promoter, and the siRNA encoding sequence. ES cells were kept under puromycin selection during 48 h following transfection. (B) Abnormal heterochromatin organization in p150CAF-1-depleted ES cells. Immunodetection of p150CAF-1 (green) and HP1α (red) in ES cells transfected with control (cont) and p150CAF-1 siRNA. siRNA expression results in efficient p150CAF-1 depletion. The right-hand image shows the merge between HP1α fluorescence and DAPI-stained DNA in blue. Scale bar = 10 μm. (C) Heterochromatin organization is not altered in p150CAF-1-depleted MEFs. Immunodetection of p150CAF-1 (green) and HP1α (red) in MEFs transfected with control (cont) or p150CAF-1 siRNA. The right-hand image shows the merge between HP1α fluorescence and DAPI-stained DNA in blue. (D) PML bodies are not altered in p150CAF-1-depleted ES cells. Immunodetection of PML (red) in ES cells transfected with control (cont) or p150CAF-1 siRNA. The right-hand image shows the merge between PML fluorescence and DAPI-stained DNA in blue.

Next, we wondered whether a similar requirement for CAF-1 could also be observed in ES cells, which are derived from the blastocyst inner cell mass. Given the early developmental arrest observed in *Chaf1a* ^−/*−*^ embryos, such cells could not be derived directly from null embryos, and we thus used an RNAi strategy (Figure 2A). p150CAF-1 knockdown in ES cells was quantified by Western blot analysis and IF. (Figure S1). p150CAF-1 RNAi-depleted ES cells displayed a phenotype very similar to the cells of *Chaf1a* ^−/*−*^ E4 mice. DAPI-dense foci were lost, and we observed diffuse HP1α and DAPI staining around the nucleoli and at the periphery of the nucleus (Figure 2B). This result indicates that p150CAF-1 is essential for nuclear organization of heterochromatin in ES cells in a similar way to early pre-implantation embryos. Surprisingly, we did not observe this severe alteration in heterochromatin organization in primary mouse embryonic fibroblasts (MEFs) following p150CAF-1 depletion by RNAi. p150CAF-1 depletion in MEFs resulted in a strong inhibition of cell proliferation (unpublished data), but did not alter the clustering of heterochromatin domains (Figures 2C and S2). Similarly, p150CAF-1 depletion in 3T3 cells did not affect heterochromatin organization [16]. These observations reveal a specific function for CAF-1 in the early embryo and ES cells.

We also wondered whether loss of p150CAF-1 in ES cells specifically affects the heterochromatin nuclear subcompartment, or whether it might result in a more global loss of nuclear organization and architecture. We used a specific antibody against the promyelocytic leukemia (PML) protein to study the fate of the PML nuclear bodies, which are well-characterized subnuclear structures [22]. We could not detect any significant difference in the distribution and aspect of PML nuclear bodies between control and p150CAF-1-depleted ES cells (Figure 2D). These data show that nuclear architecture is not globally altered following CAF-1 loss-of-function. In addition, Western blot analysis revealed that CAF-1 depletion did not result in altered levels of chromatin architectural proteins such as histone H3 and HP1α (Figure S1). Thus, CAF-1 is specifically required in early embryos and in ES cells for the proper organization of the heterochromatin subnuclear compartment.

**Figure 3:**
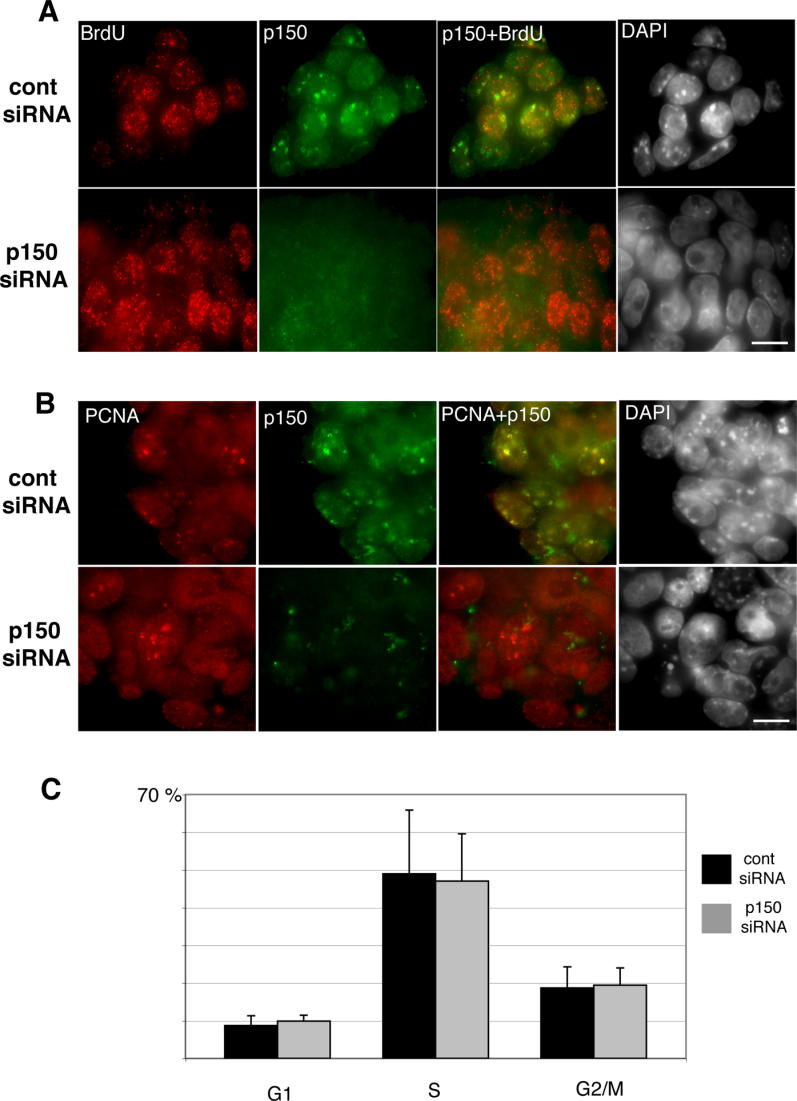
p150CAF-1 Depletion and Loss of Heterochromatin Organization Are Compatible with Active DNA Replication in ES Cells (A) ES cells were transfected with p150CAF-1 or control siRNA vectors. After 3 d under puromycin selection, cells were pulse-labeled for 10 min with BrdU and immediately analyzed by IF. No significant difference in BrdU incorporation could be detected between control and p150CAF-1-depleted cells. The right-hand image shows the merge between the p150CAF-1 and BrdU fluorescence. Scale bar = 10 μm. (B) Immunodetection of PCNA (red) revealed no significant difference in the formation of replication foci between control and p150CAF-1-depleted ES cells. Immunodetection of p150CAF-1 is shown in green and the merging of p150CAF-1 and PCNA appears in yellow. (C) Flow cytometry analysis showed a similar cell-cycle profile for control and p150CAF-1-depleted ES cells. Results are presented as the percentage of cells in each phase of the cell cycle (G1, S, G2/M), as defined by BrdU incorporation and DNA content. Data presented are the mean of three independent experiments; error bars indicate the standard deviation. All experiments were performed 3 d after transfection of the siRNA vectors.

Previous studies performed in mammalian cell lines showed that CAF-1 activity is required for S phase progression [16,23,24]. We show here that 3 d after transfection of the RNAi vector, p150CAF-1-depleted cells (identified by a complete absence of CAF-1 IF signal) appear still active for replication, as revealed by incorporation of the thymidine analog bromodeoxyuridine (BrdU) (Figure 3A), the PCNA (proliferating cell nuclear antigen) pattern (Figure 3B), and flow cytometry analysis (Figure 3C). Hence, in these cells, DNA replication persists despite the drastic changes in heterochromatin organization. However, 24 h later, ES cells cease to proliferate and die, revealing that p150CAF-1 is required for an essential cellular process, as in embryos. Altogether, our data demonstrate that loss of p150CAF-1 function in ES cells and early embryos alters first the organization of heterochromatin in the nucleus and, in a subsequent step, cell cycle and viability.

**Figure 4:**
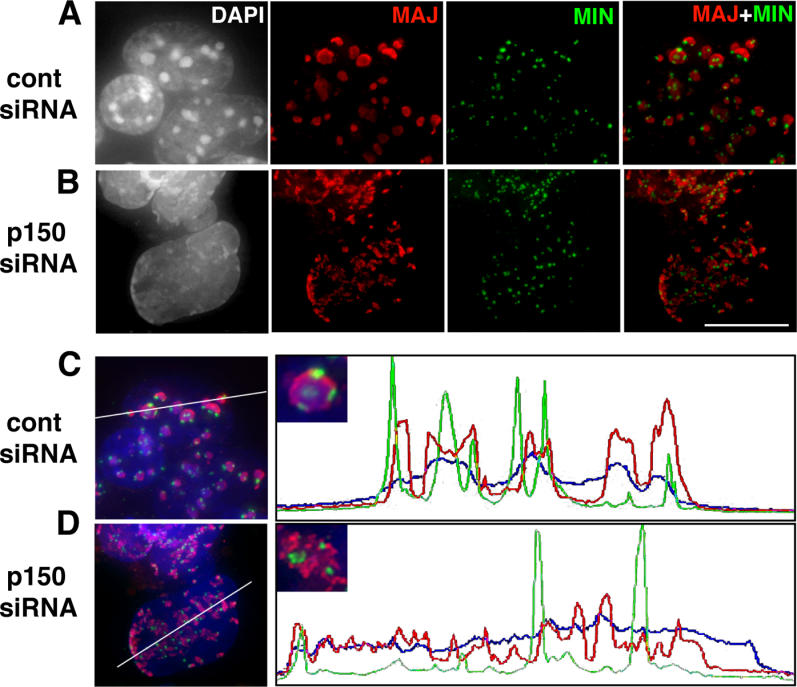
Depletion of p150CAF-1 Leads to Loss of Clustering, Altered Localization, and Decondensation of Pericentric Heterochromatin Domains Distribution of pericentric (red) and centric (green) domains was analyzed in the interphase nuclei of mouse ES cells by DNA FISH, using major satellite (pSAT) [47] and minor satellite (pMR150) [48] DNA probes, respectively. (A) In ES cells expressing control (cont) siRNA, pericentric regions from several chromosomes associate in clusters (red). These chromocenters form foci as revealed by DAPI staining (left-hand image), while centric regions (green) remain independent entities at the periphery of these domains. The right-hand image shows the merge between the pericentric and centric FISH signals. (B) The organization of pericentric domains was altered in cells expressing p150CAF-1 siRNA. Instead of forming well-defined chromocenters, pericentric domains were found either isolated or associated in heterogeneous aggregates of various sizes, often at the nuclear periphery. Scale bar = 10 μm. (C) Control ES cells. Fluorescence was quantified along a line randomly drawn across the nucleus in the merged image and data were plotted. One can distinguish clear peaks corresponding to chromocenters (red) and the condensed minor satellites (green). (D) ES cells expressing p150CAF-1 siRNA. p150CAF-1 depletion led to a lower fluorescence intensity and a broader distribution of signals corresponding to DAPI (blue) and major satellite hybridization (red, plot) while the organization of the minor satellites remained unaffected. Insets in the right-hand images show a typical chromocenter in control cells (C) and a disrupted chromocenter in p150CAF-1-depleted cells (D).

In order to better characterize the defects in heterochromatin organization, we performed two-color DNA fluorescence in situ hybridization (FISH) experiments to reveal the spatial distribution of pericentric and centric chromosomal regions, which in mouse are mainly composed of large blocks of major and minor satellite repeats, respectively [20]. Pericentric domains from different chromosomes form clusters, which are revealed by FISH as large spots that coincide with DAPI-dense foci in the interphase nucleus (Figure 4A). At the periphery of each pericentric domain, centric regions form individual entities (Figure 4A). In p150CAF-1 RNAi-depleted cells, we observed a disruption of pericentric heterochromatin clusters that coincides with the disappearance of DAPI-dense foci (Figure 4B). Individual pericentric domains from single chromosomes are now found either isolated or aggregated in a pattern less dense than the regular clusters observed in control cells (Figure 4B). Such aggregates, which are often found at the nuclear periphery, are also revealed by DAPI staining as a typical signature of p150CAF-1 loss-of-function. Quantification of fluorescence along a line randomly drawn across the nucleus revealed lower fluorescence intensity and a broader distribution of DAPI and major satellite hybridization signals in p150CAF-1-depleted cells (Figure 4C and 4D), indicating decondensation of pericentric heterochromatin domains. In contrast, centric domains showed an intensity and shape similar to control cells, suggesting that they remain unaffected by p150CAF-1 depletion (Figure 4C and 4D). In conclusion, these results show that p150CAF-1 is required for the proper condensation and clustering of pericentric heterochromatin domains.

**Figure 5:**
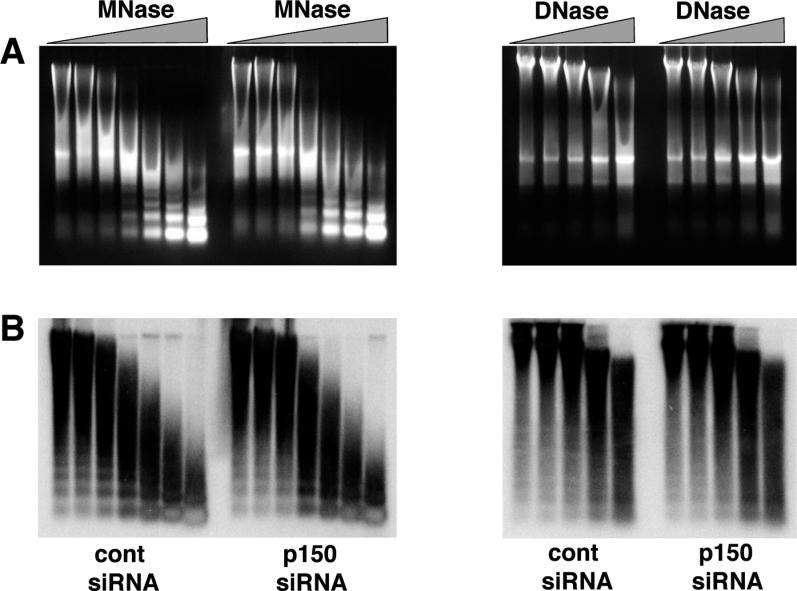
Nucleosomal Organization Is Not Altered in p150CAF-1-Depleted ES Cells Nuclei were prepared from ES cells transfected with control or p150CAF-1 siRNA vector. Nuclei were digested with increasing amounts of DNase I or MNase. (A) After digestion with the indicated nucleases, total DNA was prepared and run onto an agarose gel which was stained with ethidium bromide to reveal bulk genomic DNA. (B) The DNA was blotted onto a nylon membrane, which was then hybridized with the α-^32^P-labeled pSAT major satellite repeat probe [47].

Given the known role of CAF-1 in the deposition of histone H3.1 and H4 associated with DNA synthesis [11,12], we wondered whether this defect in higher-order chromatin organization could reflect an aberrant nucleosomal organization. Using DNase I and micrococcal nuclease (MNase) assays, we could not observe any significant difference between control and p150CAF-1-depleted ES cells at the level of bulk genome chromatin or at pericentric repeats (Figure 5). In a second series of experiments, we compared the association of histone H3 with chromatin in nuclei isolated from cells transfected with control and p150CAF-1 RNAi plasmid vectors. Nuclei were incubated in buffers with different salt concentrations ranging from 100 mM NaCl to 1 M NaCl. In all conditions, the amount of histone H3 remaining associated with chromatin was indistinguishable in control and p150CAF-1-depleted ES cells (unpublished data). Therefore, the loss of clustering and decondensation of pericentric heterochromatin are unlikely to be the consequence of severe defects in nucleosomal organization.

**Figure 6:**
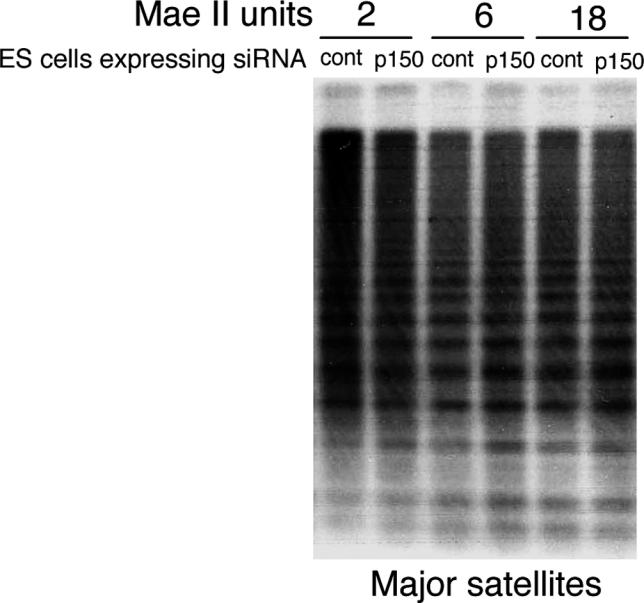
DNA CpG Methylation at Pericentric Heterochromatin Is Not Altered in p150CAF-1-Depleted ES Cells Total DNA was isolated from cells transfected with a control or the p150CAF-1 siRNA vector and digested with *Mae*II, whose recognition sequence is present in major satellite repeats. Digested DNA was run onto an agarose gel and analyzed by Southern blotting using the pSAT major satellite probe [47].

**Figure 7:**
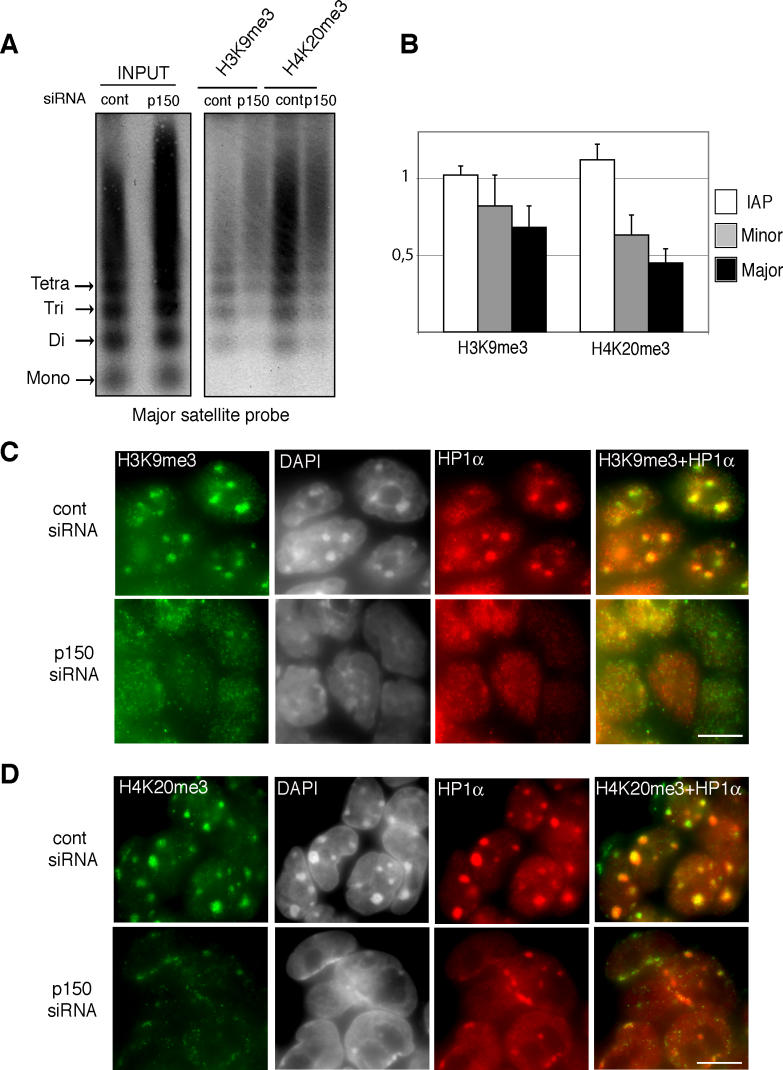
Alteration of Epigenetic Marking at Pericentric Heterochromatin in p150CAF-1-Depleted Cells (A) p150CAF-1 depletion leads to reduced H4K20me3 and H3K9me3 at pericentric heterochromatin. Enrichment of histone marks at major satellite repeats was determined by ChIP from control (cont) and p150CAF-1 (p150) siRNA-expressing ES cells. DNA prepared from the input and the antibody-bound fraction were run onto an agarose gel and analyzed by Southern blot with the pSAT major satellite repeat probe [47]. (B) Hybridization signals were quantified using an Instant Imager. After autoradiography, the membrane was stripped and rehybridized with a minor satellite probe [49]. After quantification, the membrane was stripped and rehybridized with an IAP LTR probe [50]. Results are presented as the amount of DNA immunoprecipitated from p150CAF-1-depleted ES cells divided by the DNA obtained from control cells. The figure shows the mean value and standard deviation of three independent ChIP experiments. (C and D) H3K9me3 and H4K20me3 fluorescence patterns are severely altered in p150CAF-1-depleted ES cells. Immunodetection of H3K9me3 (C, green), H4K20me3 (D, green), and HP1α (red) in control and p150CAF-1 siRNA-expressing ES cells. Merging of HP1α with H3K9me3 (C) and H4K20me3 (D) is shown in yellow. Scale bars represent 10 μm.

Our findings showed that the nuclear localization of HP1α is severely altered in *Chaf1a* ^−/*−*^ embryos and p150CAF-1-depleted ES cells. We therefore examined the status of other epigenetic marks previously shown to characterize pericentric heterochromatin. We first tested DNA CpG methylation [25] at major satellite repeats and found no significant difference between control and p150CAF-1-depleted cells (Figure 6). As the LTM7 ES cell line that we used in our RNAi experiments is a female (XX) cell line, DNA methylation is globally reduced in these cells [26]. To rule out the possibility that hypomethylation of pericentric repeats might contribute to the destabilization of heterochromatin domains in p150CAF-1-depleted ES cells, we tested our RNAi plasmid vector in a XY ES cell line, and confirmed that loss of p150CAF-1 leads to disruption of heterochromatin organization independently of the degree of DNA methylation at pericentric repeats (unpublished data). We next analyzed specific histone modifications [27] in chromatin immunoprecipitation (ChIP) experiments using native chromatin [28]. We found that pericentric DNA was immunoprecipitated with a 2-fold lower efficiency with H4K20me3 [29,30] antibodies following p150CAF-1 depletion (Figure 7A and 7B). Immunoprecipitation of minor satellite repeats in the same experiment was less affected (Figure 7B), showing that loss of p150CAF-1 function mainly affects H4K20me3 at pericentric heterochromatin. Moreover, no significant difference in immunoprecipitation efficiency was detected at intracisternal A particle (IAP) elements (Figure 7B), which are noncentromeric repeated DNA elements also enriched in H4K20me3 [31]. This result shows that p150CAF-1 contributes to normal levels of H4K20me3 at pericentric heterochromatin, but not necessarily at other loci. The H3K9me3 mark [32] was also significantly reduced in p150CAF-1-depleted cells, though to a lesser extent than H4K20me3 (Figure 7A and 7B).

In wild-type cells, most of H4K20me3 and H3K9me3 signals are present at the level of DAPI-dense domains when visualized by IF [29] (Figures 7C and 7D). H3K9me3 was severely perturbed in p150CAF-1-depleted cells, displaying a diffuse pattern similar to HP1α (Figure 7C). The H4K20me3 pattern was even more severely altered, showing both a loss of the typical dots revealed in control cells and a reduction in signal intensity (Figure 7D). Consistent with our ChIP experiments, residual H4K20me3 epitopes were detected at the level of relocated DAPI-dense material. Altogether, our results show that depletion of CAF-1 in ES cells leads to a simultaneous disruption of heterochromatin 3-D organization and an alteration in epigenetic marking.

## Discussion

Here, we provide evidence that p150CAF-1 has a crucial function during mouse early embryogenesis and in ES cells. Previous studies showed that CAF-1 is essential for the progression of DNA replication in vertebrates. In *Xenopus laevis,* p150CAF-1 function is required for the rapid cell divisions that occur during early development [33]. Similarly, CAF-1 is essential in mammalian cell lines for the progression of DNA replication [16,23,24,34]. Our findings reveal a role for CAF-1 in the nuclear organization of heterochromatin domains during early development and in ES cells. We show that in p150CAF-1-depleted ES cells, while DNA replication initially persists without significant perturbation in nucleosomal organization, severe heterochromatin organization defects can be observed. Loss of p150CAF-1 did not affect PML bodies in ES cell nuclei, showing that nuclear architecture is not generally altered. Ultimately, p150CAF-1 depletion results in the arrest of ES cell division and cell death. Given the major defects observed in heterochromatin organization, it is tempting to speculate that these defects cause the developmental arrest in the embryos and death in ES cells. The role of CAF-1 in the nuclear organization of heterochromatin includes spatial localization, condensation, and clustering of pericentric domains. This important function of CAF-1 was not revealed in primary MEFs, 3T3 cells, or human cell lines, suggesting that it is specific of pluripotent embryonic cells. Alternatively, early embryos and ES cells, whose genome is more plastic than somatic cells, might represent a particularly sensitive context in which the importance of CAF-1 in heterochromatin organization is exacerbated. In ES cells, major architectural chromatin proteins are hyperdynamic and bind relatively loosely to chromatin [35]. In this cellular context, our findings show that proper heterochromatin nuclear architecture relies on CAF-1 function. In contrast, in undifferentiated cells that are already lineage committed (such as MEFs) genome architecture might be more stable than in ES cells and thus cannot be disrupted by CAF-1 depletion. This important function of CAF-1 in pluripotent embryonic cells could be partially conserved during evolution. Indeed, in *Arabidopsis thaliana,* in which CAF-1 is not essential for viability [36], loss of CAF-1 is associated with a mild alteration in chromocenter size [37]. This phenotype is somehow reminiscent of loss of CAF-1-mediated defects in heterochromatin organization in early mouse embryos. In mouse ES cells, we show that CAF-1 is also required for regular levels of H4K20me3 at pericentric repeats, but not at IAP elements, which are repeated sequences spread all over the mouse genome. These data suggest that proper nuclear organization and epigenetic marking of pericentric heterochromatin are coupled in ES cells. Alternatively, 3-D organization of heterochromatin and epigenetic marking might represent two independent functions of CAF-1.

The variable requirement for CAF-1 across species might reflect the acquisition of new properties affecting the function of histone H3 variants during evolution of eukaryotes. CAF-1 is essential in mammals but dispensable in Saccharomyces cerevisiae [38]. Interestingly, the budding yeast genome does not contain heterochromatin regions equivalent to those of higher eukaryotes, and several hallmark features of heterochromatin such as di- and trimethylation at H3K9, H3K27, and H4K20 are not observed in the silent chromatin of *S. cerevisiae.* In mammals, H3K9 dimethylation is predominantly associated with H3.1, but not with H3.3, which carries modifications typically found in transcribed chromatin regions [39]. Outside of the centromeric H3 variant, S. cerevisiae possesses only H3.3, which is expressed and incorporated into chromatin in a replication-independent fashion in higher eukaryotes [12]. These data suggest a specific role for H3.1 in heterochromatin regions. We show here that CAF-1, which is a H3.1 replication-dependent loading complex [11], is specifically required for the nuclear organization of pericentric heterochromatin in embryos and ES cells. One possibility would be a scenario in which incorporation of H3.1 into chromatin is required for the proper nuclear organization of constitutive heterochromatin in an embryonic context. While this has to be addressed experimentally, it is worthwhile to mention an alternate possible mechanism that might explain the disruption of heterochromatin domains in p150CAF-1 null embryos and ES cells. CAF-1 could be required for the loading, into heterochromatin, of an interacting partner required for the clustering of pericentric domains. Following one or two rounds of DNA replication in the absence of p150CAF-1, heterochromatin would become deprived of this molecule, which would cause the disruption of its 3-D organization. A potential candidate is the HP1 protein, which interacts with CAF-1 [13,16].

In agreement with a recent report [40], our analysis of heterochromatin in pre-implantation embryos has revealed drastic changes in nuclear organization between the two-cell and blastocyst stages. Cloning experiments in mouse revealed that the nuclear organization of heterochromatin in ES cell nuclei was quickly reverted into the one-cell stage-specific form after nuclear transfer [40]. Hence, remodeling of heterochromatin 3-D organization parallels nuclear reprogramming toward an early embryonic status in cloned embryos. This drastic reorganization of heterochromatin organization is similar to that observed in p150CAF-1-depleted ES cells, and in developmentally arrested 16-cell stage *Chaf1a^−/−^* embryos. These data show that CAF-1 is involved in setting up proper heterochromatin architecture during the first cell divisions of embryonic life. Given the importance of nuclear organization in the control of gene expression [21,41], this finding suggests that CAF-1 contributes to the coordinated programs of gene expression during early embryogenesis. Our findings open new perspectives in the understanding of chromatin dynamics during early development and in pluripotent embryonic cells. While most studies focused on the role of DNA methylation and histone modifying enzymes [3], our data point to the importance of the chromatin assembly machinery. We show that in addition to assembling nucleosomes, CAF-1 provides spatial and epigenetic information to heterochromatin domains in early embryos and ES cells.

## Materials and Methods

### Generation of *Chaf1a* mutant mice and embryos.

Using PCR, we amplified two genomic fragments (about 3 kb each) flanking *Chaf1a* exon 3. These DNA fragments were assembled by conventional cloning with the neomycin and DT (diphtheria toxin) cassettes, as described in Figure 1A. The construct was transfected in ES cells by electroporation. We identified recombinant ES cells carrying the mutant *Chaf1a* ^tm1Ger^ (abbreviated *Chaf1a* ^−^ in the manuscript) allele by Southern blot (Figure 1A). We derived *Chaf1a* ^+/*−*^ mice by injecting recombined ES cells into C57BL/6N blastocysts. E4 embryos obtained from the intercross of *Chaf1a* ^+/*−*^ mice were genotyped by nested-PCR amplification. Embryos were collected in a PCR reaction mix containing oligonucleotide primers 1, 2, and 3 (Figure 1A) that amplify the wild-type and mutant alleles. After 30 cycles of amplification, 1 μl of each PCR reaction was used in a second round of PCR amplification with a new set of oligonucleotide primers. After 30 cycles of amplification, PCR reactions were run onto an agarose gel, which revealed the presence of the wild-type (150 bp) and recombinant (200 bp) alleles.

### P150CAF-1 depletion by RNAi. 

The RNAi plasmid vector that we used in this study contains the mouse H1 promoter and a puromycin selection gene (SB and MG, unpublished data). We characterized in detail the properties of this new vector using a GFP target gene and FACS: Extinction of target genes in ES cells is highly efficient in 70% of the cells, of intermediate efficiency in 15%, and inefficient in the remaining 15% of cells analysis. The sequence of p150CAF-1 siRNA (short interfering RNA) duplex [16] was cloned into this plasmid. The control vector expressed a siRNA that targets GFP RNA degradation. ES cells were transfected by electroporation with the RNA plasmid vector, seeded onto gelatin-coated slides, and cultured for 24 h in the absence of selection. Puromycin (2 μg/ml) was added to the culture medium and cells were cultured for an additional 48-h period. Using the p150CAF-1 RNAi vector, IF microscopy quantification reveals a complete CAF-1 depletion in most cells (Figure S1B). Western blot analysis (Figure S1A) reveals residual p150CAF-1 expression, as expected from the 15% of cells that do not inhibit the target gene.

MEFs (passage 3, 90% confluence) were transfected during 4 h with the RNAi plasmid vector using Lipofectamine 2000 (Invitrogene, Carlsbad, California, United States) according to manufacturer's conditions. Cells were trypsinized, plated at 1/3 dilution, and cultured for 48 h in the presence of puromycin.

### Cell lines.

AT-1 ES cells [42] (a gift of M. Vernet) were used for gene targeting. LTM7 ES cells were used in all RNAi experiments. We derived this cell line from (C57BL/6 × 129) F1 females bred with C3H/HeJ males. LTM7 are XX ES cells, competent for germ line transmission. Primary MEFs were derived from E13 embryos as described in [43].

### Immunofluorescence.

Cells were fixed for 20 min in PBS with 4% paraformaldehyde, and immunodetection was performed as previously described [44]. E4 embryos were collected and treated with tyrode acid to remove the zona pellucida, deposited onto microscope slides, and processed for immunostaining as described for ES cells. Antibodies anti-HP1α (2HP1H5, Euromedex, France), anti-H4K20me3 (Abcam, Cambridge, United Kingdom), anti-BrdU (DakoCytomation, Glostrup, Denmark), anti-PCNA (DakoCytomation), and anti-PML (Upstate Biotechnology, Lake Placid, New York, United States) were all used at 1/1000 dilution. Antibodies anti-mouse p150CAF1 [16] and anti-H3K9me3 (Upstate Biotechnology) were used at 1/250 and 1/500, respectively. All secondary antibodies were purchased from Molecular Probes (Sunnyvale, California, United States).

### Western blot analysis.

Cells were lysed in Laemli buffer and run onto a 4%–12% SDS PAGE gradient gel. We used antibodies against p150CAF-1 ([16]; dilution 1/500), HP1α (2G9, Euromedex; 1/500), Histone H3 (Abcam; 1/500), H3K9me3 (Upstate; 1/500) and β-actin (Upstate; 1/40 000).

### DNA FISH.

Probes were described previously [20]. Biotin-16-dUTP or Digoxigenin-11-dUTP (Roche, Basel, Switzerland) labeled probes were generated by nick translation (Roche) and FISH performed as described [20]. Image acquisition was performed with the Deltavision RT microscope (100×, 1.4 NA objective), images were deconvoluted, and fluorescence profiles measured along an arbitrary line using SoftWorx.

### Nuclei preparation, nuclease digestion, and biochemical analysis of chromatin.

ES cells were incubated on ice for 10 min in buffer 1 (15 mM Tris-HCl [pH 7.5], 0.3 M sucrose, 60 mM KCl, 15 mM NaCl, 5 mM MgCl2, 0.1 mM EGTA) with 0.15% IGEPAL (Sigma, St. Louis, Missouri, United States). Nuclei were purified by centrifugation (10,000 × *g* for 30 min, at 4 °C) on sucrose cushions (buffer 1 with 1.2 M sucrose) and resuspended in nuclease buffer (50 mM Tris-HCl [pH 7.5], 20 mM NaCl, 0.32 M sucrose, 4 mM MgCl_2_, 1 mM CaCl_2_). About 2.10^6^ nuclei were incubated with increasing quantities of MNase (0.04–1.6 units) or DNase I (0.25–16 units). Digestion time at 37 °C was 10 min for MNase and 2 min for DNase I. Digestions were stopped by adding SDS to 1% and EDTA to 50 mM. DNAs were prepared by proteinase K digestion followed by phenol-chloroform extraction and isopropanol precipitation. To test the association of histone H3 with chromatin in control and p150-depleted cells, isolated nuclei were incubated on ice for 30 min in buffer 2 (50 mM Hepes [pH7.9], 20% Glycerol, 3 mM MgCl2, 0.1% IGEPAL, 0.5 mM DTT, 0.5 mM PMSF) supplemented with either 0.1 M, 0.3 M, 0.45 M, 0.7 M, or 1 M NaCl. After centrifugation at 15,000 *g,* pellets and supernants were analyzed by Western blot using a histone H3 antibody (Abcam).

### ChIP.

We prepared native chromatin fragments of two to six nucleosomes in length as described [28]. 5 μg of chromatin were incubated overnight with 10 μl of commercial antibodies. After incubation with protein-G sepharose and three washes, immunoprecipitated DNA was purified, sized onto an agarose gel, and analyzed by Southern blot. Hybridization signals were quantified using an Instant Imager (PerkinElmer, Wellesley, California, United States). Antibodies specific for H4K20me3 were purchased from Abcam. Antibodies for H3K9me3 were purchased from both Abcam and Upstate Biotechnology and gave similar results.

For Southern blot analysis, we used a 240-bp EcoRI/BamHI fragment from pSAT (major satellite probe) [45–47] and a 360-bp EcoRI/HindIII fragment from R198, corresponding to three copies of the 120-bp minor satellite repeat [49]. Probes used in FISH experiments were described in [46–48]. The probe for IAP elements was described in [50].

## Supporting Information

Figure S1 Analysis of p150CAF-1 Depletion by RNAi with Western Blot and IF (A) Western blot analysis of ES cells transfected with control (cont) or p150CAF-1 (p150) RNAi plasmid vectors. β-actin was used as a loading control. Amount of cells used in each lane is indicated. p150CAF-1 knockdown does not significantly affect HP1α, histone H3, or H3K9me3 levels.
(B) IF analysis. ES cells transfected with control (cont) or p150CAF-1 (p150) siRNA vector were immunostained with p150CAF-1 antibody (green) and DAPI (blue). Scale bar = 5 μm. Fluorescence was quantified along a line drawn across the nucleus and data were plotted, revealing efficient p150CAF-1 depletion by RNAi.
The apparent difference in the efficiency of CAF-1 knockdown revealed by Western blot and IF likely reflects the observation that the RNAi plasmid vector used in this study is efficient in 85% of the transfected ES cell population, whereas 15% of cells display no inhibition of the target gene (see Materials and Methods).
(1.3 MB TIF)

Figure S2 CAF-1-Depleted Embryonic Fibroblasts Active for DNA Replication Do Not Display an Altered Heterochromatin Nuclear Architecture Immunodetection of p150CAF-1 (green) and BrdU (red) in MEFs transfected with control (cont) or p150CAF-1 RNAi vectors. MEFs were pulse-labeled for 1 h with BrdU prior to fixation and IF analysis. The p150 + BrdU image shows the merge between p150CAF-1 and BrdU fluorescence. DAPI staining is shown in the right-hand panel. The cytoplasmic CAF-1 IF pattern is non-specific.
(343 KB TIF)

## Abbreviations

BrdU: bromodeoxyuridine
CAF-1: chromatin assembly factor 1
ChIP: chromatin immunoprecipitation
E4: embryonic day 4
ES: embryonic stem
FISH: fluorescence in situ hybridization
IAP: intracisternal A particle
IF: immunofluorescence
MEF: mouse embryonic fibroblast
PCNA: proliferating cell nuclear antigen
PML: promyelocytic leukemia
RNAi: RNA interference
siRNA: short interfering RNA
